# Parental separation and adult psychological distress: an investigation of material and relational mechanisms

**DOI:** 10.1186/1471-2458-14-272

**Published:** 2014-03-23

**Authors:** Rebecca E Lacey, Mel Bartley, Hynek Pikhart, Mai Stafford, Noriko Cable

**Affiliations:** 1 Department Epidemiology & Public Health, UCL, 1-19 Torrington Place, WC1E 6BT London, UK; 2 MRC Unit for Lifelong Health & Ageing, 33 Bedford Place, WC1B 5JU London, UK

**Keywords:** Divorce, Material disadvantage, Parent–child relationships, Psychological distress, British cohort study

## Abstract

**Background:**

An association between parental separation or divorce occurring in childhood and increased psychological distress in adulthood is well established. However relatively little is known about why this association exists and how the mechanisms might differ for men and women. We investigate why this association exists, focussing on material and relational mechanisms and in particular on the way in which these link across the life course.

**Methods:**

This study used the 1970 British Cohort Study (n = 10,714) to investigate material (through adolescent and adult material disadvantage, and educational attainment) and relational (through parent–child relationship quality and adult partnership status) pathways between parental separation (0–16 years) and psychological distress (30 years). Psychological distress was measured using Rutter’s Malaise Inventory. The inter-linkages between these two broad mechanisms across the life course were also investigated. Missing data were multiply imputed by chained equations. Path analysis was used to explicitly model prospectively-collected measures across the life course, therefore methodologically extending previous work.

**Results:**

Material and relational pathways partially explained the association between parental separation in childhood and adult psychological distress (indirect effect = 33.3% men; 60.0% women). The mechanisms were different for men and women, for instance adult partnership status was found to be more important for men. Material and relational factors were found to interlink across the life course. Mechanisms acting through educational attainment were found to be particularly important.

**Conclusions:**

This study begins to disentangle the mechanisms between parental separation in childhood and adult psychological distress. Interventions which aim to support children through education, in particular, are likely to be particularly beneficial for later psychological health.

## Background

Divorce rates have increased rapidly in Great Britain, like other Western countries, over recent decades and are known to be a risk factor for poorer child and adult outcomes. Psychological distress is one frequently-investigated adult outcome; children who experience parental separation (in this paper defined as the breakdown in partnership between parents regardless of marital status) are more likely to report symptoms of depression and anxiety in adulthood e.g. [1-6]. However relatively little is known about how parental separation contributes to poorer psychological distress and how these mechanisms might differ for men and women.

This study distinguishes two main mechanisms linking parental separation to psychological distress in adulthood: material and relational. Divorce and separation are known to result in a decline in living standards. Where the child’s custodial family is headed by a single mother there is a particular risk of material disadvantage during adolescence [7,8]. This may be because of the costs of running two households, legal fees, reliance on benefits, a single-parent income, and the reduced earning power of women due to increase time in domestic work or unequal pay in the workplace. Also parental separation has been linked to reduced educational attainment in many studies e.g. [9-11]. This may be due to lower parental support, lack of understanding or support from schools, and the financial need for children to work rather than remain in education. Educational attainment may be an important means through which material disadvantage persists across the life course; children who are more materially disadvantaged in childhood are known to have lower educational attainment [12], which in turn increases the chances they will remain materially disadvantaged as an adult. In turn both material disadvantage in adulthood and educational attainment are associated with psychological distress [13,14].

It is therefore possible that material pathways are involved in mediating the association between parental separation and psychological distress. Ross and Mirowsky [9] found the association between parental separation and adult depression was partially mediated by education, as well as occupational status and economic hardship. Additionally Amato [15] found that the association between parental absence due to divorce and adult depression was partially explained upon adjustment for educational attainment. However many studies are cross-sectional and prone to misreporting of childhood exposures. Also many studies employ simple regression methods which do not allow for the explicit modelling of mechanisms across the life course.

Material factors are also likely to be involved pre-separation as known risk factors for divorce [16,17]. Therefore failure to take account of prior material disadvantage may result in misleading conclusions. Mandemakers et al. [18] consider early life disadvantage to be a modifier of the association between parental divorce and psychological distress, however in our study we argue that material disadvantage is an important pathway or explanatory variable between parental separation and adult psychological health. We therefore controlled for indicators of disadvantage prior to parental separation in order to better assess whether those who experienced parental separation are more disadvantaged later, over and above their level of disadvantage prior to separation. Also this approach helps us to assess whether adolescent material disadvantage is a result of parental separation, rather than a continuation of life course socioeconomic disadvantage.

There is also evidence that relational pathways may be important. For example, parental separation can lead to reduced quality of parent–child relationships. Bowlby’s attachment theory [19] describes how the experience of parental loss and conflict can disrupt the secure bond between the child and its parents. There is evidence from previous studies to show that this is the case e.g. [20,21]. Parent–child relationships can have long-term influences on the health in later life [22,23].

Also children from separated families are more likely to experience the breakdown of their own adult partnerships [24,25]. This is thought to be due to increased marital problems, greater acceptance of divorce and exposure to less positive marital interactions as a child [26-28]. In turn adult partnership breakdown is a known risk factor for psychological distress e.g. [29].

A relational pathway between parental separation and adult psychological distress has previously been partially investigated, mainly focusing upon parent–child relationships [30,31] and adult partnerships [32], showing both to be important. However previous analyses of relational mechanisms have not combined these with important material mechanisms and have treated them as two distinct and unrelated pathways, which is unlikely to be the case. This study looks at how material and relational mechanisms might interlink across the life course.

**Figure 1:**
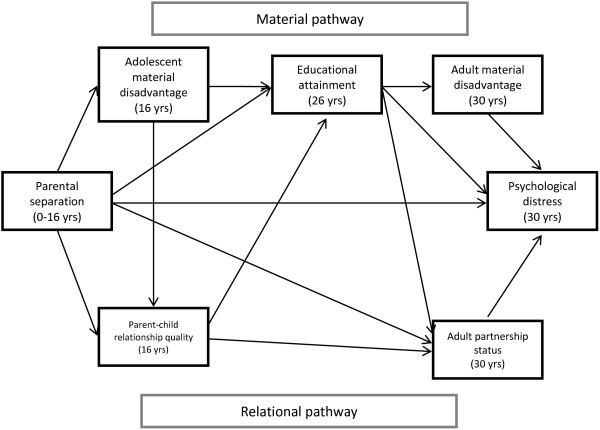
Conceptual model of material and relational pathways linking parental separation and adult psychological distress.

Based on previous studies we propose a model of material and relational pathways linking parental separation in childhood and adult psychological distress (Figure 1). By taking a path analysis approach we were able to additionally investigate how material and relational factors relate to each other across the life course, as it is unlikely that they operate in isolation from each other. For example, based on the Family Stress Model [33] a link is proposed between adolescent material disadvantage and parent–child relationship quality. We also examine whether parent–child relationship quality influences educational attainment, as suggested by other work [34,35]. Finally a path is proposed between educational attainment and partnership status, as those leaving school earlier are more likely to marry younger and are more likely to divorce [36]. These linkages between material and relational factors are consistent with Amato’s ‘divorce-stress-adjustment’ [37] whereby parental relationship problems potentially unfold into a series of negative consequences for children across emotional, interpersonal and academic domains.

Gender differences in the association between parental separation and adult psychological distress, and the pathways involved, are also not well established. Some studies find no gender differences in association between separation and adult depression and anxiety e.g. [30,38]. However some found women were more adversely affected, perhaps partly because they are more likely to emotionally support a parent and to assume a parental role post-separation [2,3,39]. These inconsistencies suggest that men and women may be affected differently and there is some indication that the mechanisms linking parental separation and psychological distress differ by gender [30,40,41]. For example previous work has shown that there has been more priority for boys to remain in education, compared to girls, following parental separation [42,43]. Also girls have been found to report poorer quality post-separation parent–child relationships than boys [30,40]. These studies would therefore suggest that women may be more adversely affected with regards to material and relational factors. However this needs to be explored further using an appropriate statistical method which allows for the investigation of mechanisms, and whether these differ for men and women.

## Methods

### Sample

This study uses the 1970 British Birth Cohort, which aimed to recruit all babies born during a single week of 1970 in Great Britain (achieved sample = 16,571, 95.9% of target). Follow-up data were collected at 5, 10, 16, 26, 30, 34, and 38 years. In this study data from birth through 30 years were used. All measures used were prospectively-collected therefore limiting bias due to recall problems. The longitudinal data, along with the statistical approach, make this study appropriate for investigating pathways acting across the life course. Data are open access for non-commercial use. Informed consent was collected from the parents of the cohort member in childhood and from the cohort member themselves in adulthood. Ethical approval was obtained from the National Research Ethics Advisory Panel.

### Measures

#### Parental separation

Parental separation due to partnership breakdown was derived from information on who the cohort member’s parental figures were at each survey between birth and age 16. Children whose mothers were not in a partnership at birth were excluded from the analysis (n given in missing data section below) to ensure that the separation had occurred during the child’s lifetime, not before the birth.

#### Psychological distress

Psychological distress was measured at age 30 using the Malaise Inventory, developed by Rutter [44], comprised of 24 items which cover emotional disturbance and somatic symptoms. Cronbach’s alpha for the scale was 0.80 representing a high level of internal consistency. The score was positively-skewed and was therefore log-transformed.

#### Material pathways

Three material factors were included – adolescent material disadvantage, educational attainment and adult material disadvantage. The indicators of material disadvantage chosen reflect longer-term disadvantage, compared to frequently used measures such as income and work status, which may only capture conditions at a single point in time. The hypothesis in this study is that parental separation, and other variables in our model, influence longer-term living stands, and in turn these influence psychological distress. Adolescent material disadvantage was measured at age 16 and is comprised of 5 household-level measures reported by the mother – housing tenure (owns/mortgage, social housing, privately-rented, other), overcrowding (<1.5 persons per room, ≥1.5 persons per room), financial hardship in the past year (0 = no, 1 = yes), access to basic household amenities (created by summing responses for individual amenities – access to a bathroom, indoor lavatory and hot water supply, each coded as 0 = sole use, 1 = shared use or no access, therefore the total score ranged from 0 = sole use to 3 = no/shared access to all) and receipt of benefits associated with disadvantage (e.g. supplementary benefit or unemployment benefit, 0 = does not receive, 1 = benefit receipt). These variables were combined using multiple correspondence analysis and the first component prior to rotation was taken. This variable is referred to as “material advantage” in the results tables as a higher score equates to more advantage and a lower score to less advantage.

Educational attainment was measured as the highest qualification achieved by age 26 years and was coded as follows: 0 = no qualifications, 1 = CSE 2-5/O-level (qualifications achieved at age 16), 2 = A-level (qualifications achieved at age 18), 3 = higher qualifications and degrees. Adult material disadvantage was indicated at age 30 by housing tenure (coded the same as at age 16) and occupational social class measured using the Registrar General’s Social Class schema (0 = professional (I), 1 = managerial/technical (II), 2 = skilled non-manual (IIINM), 3 = skilled manual (IIIM), 4 = semi-skilled manual (IV), 5 = unskilled manual (V)).

#### Relational pathways

Parent–child relationship quality was measured at age 16 using relevant items from an adapted form of the Parental Bonding Instrument. Three items- ‘my parents are understanding’ , ‘my parents are loving/caring/look after me’ and ‘my parents are helpful/good in a crisis’ (all responses 0 = does not apply, 1 = applies) were combined into a factor score using weights derived from factor analysis. Partnership status was ascertained at age 30 taking the following categories: 0 = cohabiting, 1 = single, 2 = married, 3 = remarried, 4 = separated/divorced. Those who were widowed (n = 6) were included in the married category as this form of partner loss was not caused by relationship breakdown. Malaise scores for these participants did not significantly differ from other partnership groups.

#### Covariates

Father’s social class at the birth of the cohort member (coded as above) was controlled for as an indicator of disadvantage prior to parental separation. Mother’s education (stayed at school beyond minimum school leaving age, or left at or before minimum age) and age at birth of cohort child were also included as covariates.

#### Statistical analysis

Data were multiple imputed using chained equations, creating 20 imputed datasets to account for differential attrition. The imputation model included all analysis variables, variables predictive of missingness (e.g. markers of social disadvantage) and variables likely to provide useful information for filling in gaps (e.g. the same measures from a preceding or subsequent survey). Von Hippel’s [45] approach of multiple imputation then deletion was taken, imputing information for those with complete data on psychological distress at age 30. 11,261 participants were interviewed at age 30 (88% of target), 11,101 (98.6%) of these had complete information on psychological distress. 387 participants had mothers who were not partnered at their birth and were therefore excluded from our analysis. Therefore our final sample was 10,714. Table 1 shows a comparison of observed and imputed data. The first column entitled ‘missing’ reports the percentage of participants who were missing data for that variable. The second column entitled ‘observed’ reports the distribution of all participants who had information on that variable. The third column, ‘imputed’ , shows the distribution of sample participants following the imputation. This table indicates that the imputation has been implemented satisfactorily, as observed and imputed data are similar for all variables.

**Table 1:** Characteristics of the study sample and comparison of observed and imputed data (N = 10,714)

				**Imputed**	
	**Missing (%)**	**Observed (%)**^**a**^	**Imputed (%)**^**a**^	**Parental separation (%)**	**No parental separation (%)**
Psychological distress (30 yrs)					
Median [IQR]	0^b^	3 [1-5]	3 [1-5]	3 [1-6]	2 [1-5]
Parental separation (0–16 yrs)					
No separation	41.1	79.8	79.8	-	-
Separation		20.2	20.2	-	-
Gender (0 yrs)					
Men	8.0	48.6	48.9	49.3	48.7
Women		51.4	51.1	50.7	51.3
Adolescent material advantage (16 yrs)					
Mean [SD]	34.1	0.01 (1.00)	−0.04 (1.03)	−0.61 (1.19)	0.11 (0.93)
Educational attainment (26 yrs)					
No qualifications	35.7	4.8	6.0	9.5	5.1
CSE 2-5/O-level		58.9	60.7	67.2	59.0
A-level		10.8	10.2	8.0	10.8
Higher qualification/degree		25.5	23.1	15.4	25.1
Adult social class (30 yrs)					
Professional (I)	18.2	6.4	6.1	4.3	6.6
Managerial/technical (II)		34.9	34.0	29.8	35.1
Skilled non-manual (IIINM)		24.8	24.9	24.5	25.0
Skilled manual (IIIM)		20.5	21.0	24.9	20.0
Semi-skilled manual (IV)		10.8	11.1	13.1	10.6
Unskilled manual (V)		2.7	2.8	3.4	2.7
Adult housing tenure (30 yrs)					
Own/mortgage	0.9	65.1	65.0	57.5	67.0
Social housing		13.9	13.9	20.0	12.3
Privately-rented		12.0	12.0	14.1	11.4
Other		9.1	9.1	8.4	9.3
Parent–child relationship quality (16 yrs)					
Mean [SD]	54.2	−0.02 (0.77)	0.01 (0.75)	0.09 (0.77)	−0.01 (0.75)
Partnership status (30 yrs)					
Cohabiting	0.01	21.5	21.5	23.1	21.1
Single		28.2	28.2	29.0	27.9
Married		42.3	42.3	38.4	43.3
Remarried		1.6	1.6	1.9	1.5
Separated/divorced		6.5	6.5	7.6	6.2
Father’s social class (0 yrs)					
Professional (I)	8.4	5.4	5.4	3.3	6.0
Managerial/technical (II)		12.5	12.5	10.0	13.1
Skilled non-manual (IIINM)		13.1	13.0	10.4	13.7
Skilled manual (IIIM)		46.4	46.4	47.6	46.1
Semi-skilled manual (IV)		14.1	14.1	16.3	13.5
Unskilled manual (V)		8.5	8.7	12.4	7.7
Mother’s education (0 yrs)					
Stayed beyond minimum age	8.6	35.9	36.1	31.9	37.2
Left at or before minimum age		64.1	63.9	68.1	62.8
Mother’s age (0 yrs)					
Mean yrs [SD]	8.0	26.2 (5.30)	26.2 (5.32)	24.3 (4.8)	26.7 (5.3)

^a^Only %s are given as Ns vary across the 20 imputed datasets. ^b^No participants have missing values on psychological distress as we only impute and analyse data on those with complete information on this variable.

Abbreviations: *A-level* Advanced level, *CSE* Certificate of Secondary Education, *O-level* ordinary level, *SD* standard deviation, *IQR* interquartile range.

Path analysis was used to model material and relational mechanisms over the life course for men and women separately, controlling for covariates. This method allows for the analysis of temporally-ordered pathways across time. The model was further refined by removing statistically non-significant associations, starting with that closest to a p value of one. The model fit statistics were inspected following each refinement to ensure that the model was more appropriate. The model was developed by inspecting the modification and model fit indices. Model fit indices used were the Root Mean Square Error of Approximation (RMSEA), Comparative Fit Index (CFI) and Tucker-Lewis Index (TLI). The effect of parental separation was decomposed into ‘direct’ and ‘indirect’ effects. This is useful for assessing how much of the association between parental separation and psychological distress was explained by material and relational pathways. Standardised coefficients were estimated. All analyses were conducted using MPlus v.6.1 [46].

### Results

Table 1 shows the characteristics of the sample. Focussing on the imputed data, 20.2% of the sample experienced parental separation by age 16. The median number of Malaise symptoms reported was 3 (8 symptoms and above is taken to be indicative of psychological distress [47]). Median psychological distress scores were higher for women and those who experienced parental separation. Those who experienced parental separation were less materially advantaged at age 16, had lower educational qualifications by age 26, were in less advantaged adult social classes and were less likely to own their own homes at age 30. In addition those who experienced parental separation reported poorer quality parent–child relations and were more likely to be single, cohabiting or divorced at age 30 years. Parental separation was more common amongst families in which the occupational social class of the father was less advantaged, mothers were younger and did not stay at school beyond the minimum school-leaving age. Controlling for all covariates, parental separation was associated with increased psychological distress (β = 0.13, 95% CI: 0.08, 0.19, p < 0.001) and this did not differ for men and women (p = 0.963).

### Material and relational pathways

**Figure 2:**
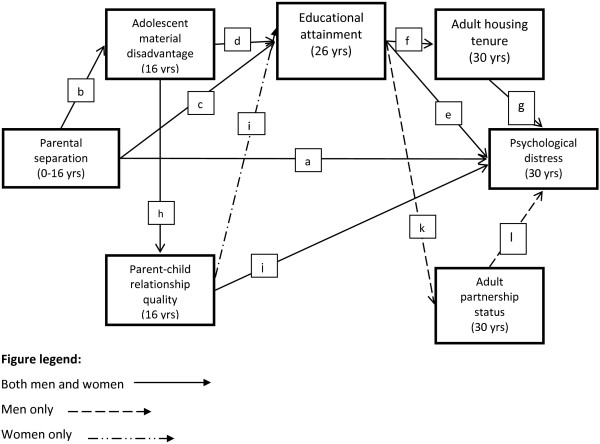
The final model of material and relational pathways linking parental separation and adult psychological distress.

Despite no evidence for gender differences in the overall association between parental separation and adult psychological distress, the mechanisms acting across the life course were different. For instance some pathways were statistically significant for men but not for women. Figure 2 and Table 2 show the results for the final path model.

**Table 2:** Final model of material and relational pathways between parental separation and adult psychological distress by gender

		**Men**		**Women**	
**Path**	**Association**	**β**	**P value**	**β**	**P value**
a	Parental separation → psychological distress	0.059	0.002	0.036	0.014
b	Parental separation → adolescent material advantage	−0.295	<0.001	−0.298	<0.001
c	Parental separation → educational attainment	−0.071	0.001	−0.087	<0.001
d	Adolescent material advantage → educational attainment	0.245	<0.001	0.227	<0.001
e	Educational attainment → psychological distress	−0.116	<0.001	−0.113	<0.001
f	Educational attainment → housing tenure (own/mortgage)	Ref		Ref	
	Educational attainment → housing tenure (social housing)	−0.230	<0.001	−0.304	<0.001
	Educational attainment → housing tenure (private renting)	0.050	0.036	0.023	0.489
	Educational attainment → housing tenure (other)	−0.031	0.248	0.008	0.699
g	Housing tenure (own/mortgage) → psychological distress	Ref		Ref	
	Housing tenure (social housing) → psychological distress	0.077	<0.001	0.077	<0.001
	Housing tenure (private renting) → psychological distress	0.053	0.001	0.034	0.019
	Housing tenure (other) → psychological distress	0.033	0.037	0.025	0.086
h	Adolescent material advantage → poor parent–child rel. qual.	−0.065	0.002	−0.085	<0.001
i	Poor parent–child relationship quality → educational attainment			−0.067	<0.001
j	Poor parent–child relationship quality → psychological distress	0.063	0.008	0.093	<0.001
k	Educational attainment → Partnership status (married)	Ref		^a^	
	Educational attainment → partnership status (cohabiting)	0.008	0.863		
	Educational attainment → partnership status (remarried)	−0.002	0.983		
	Educational attainment → partnership status (single)	−0.077	0.003		
	Educational attainment → partnership status (div/sep)	−0.050	0.118		
l	Partnership status (married) → psychological distress	Ref		^a^	
	Partnership status (cohabiting) → psychological distress	−0.013	0.732		
	Partnership status (remarried) → psychological distress	0.052	0.933		
	Partnership status (single) → psychological distress	0.035	0.012		
	Partnership status (div/sep) → psychological distress	−0.007	0.781		
Total effect		0.063 (100%)		0.115 (100%)	
Direct effect		0.042 (66.7%)		0.046 (39.1%)	
Indirect effect		0.021 (33.3%)		0.069 (60.0%)	

Model fit statistics: Men: CFI = 0.811, TLI = 0.730, RMSEA = 0.011. Women: CFI = 0.909, TLI = 0.803, RMSEA = 0.045.

R^2^ for psychological distress, men = 0.044; women = 0.039.

^a^Pathways removed in final model due to statistical insignificance.

Focusing firstly on men, parental separation was associated with lower material advantage in adolescence and lower educational attainment. Those who were more advantaged in adolescence tended to have higher educational qualifications. In turn those with higher qualifications were less likely to be living in social housing at age 30. Living in social housing and lower educational attainment were both associated with increased psychological distress. Pathways operating via adult social class were found to be statistically insignificant and were therefore removed from the model.

The relational pathways were slightly different to those predicted. There was no statistically significant link between parent–child relationship quality and adult partnership status, or between parental separation and adult partnership status. However an additional pathway between parent–child relationship and men’s psychological distress was added in response to modification indices, suggesting that positive parent–child relationships were associated with lower psychological distress.

The final model highlights the importance of considering material and relational pathways as overlapping domains. Relational factors were found not to directly mediate the relationship between parental separation and psychological distress but to interact with material factors across the life course. For example, adolescent boys who were more materially advantaged reported better quality parent–child relationships. Also men who had higher educational attainment were less likely to be single at age 30. Material and relational mechanisms in combination explained 33% of the ‘total effect’ of parental separation on psychological distress in men. But even after accounting for all material and relational factors, parental separation in childhood was independently associated with increased psychological distress for men. This shows that other factors were involved which were not included in the material or relational pathways as we measured them.

Table 2 also shows the results for women. Similar to men, parental separation was associated with increased adolescent material disadvantage and reduced educational attainment. Having higher qualifications was associated with being less likely to live in social housing and reduced psychological distress. As in men, social class at age 30 was not associated with psychological distress and was not on the pathway between parental separation and psychological distress.

The relational pathway in women was quite different to that for men; paths through adult partnership status were not statistically significant. This difference appears to be largely driven by relatively higher levels of psychological distress in single men compared to single women. For women there appeared to be more overlap between material and relational mechanisms as parent–child relationship quality was significantly related to educational attainment, suggesting that those reporting poorer quality relationships with their parents tended to have lower educational qualifications in early adulthood. 60% of the ‘total effect’ of parental separation on psychological distress was explained by material and relational mechanisms and the way these interacted across the life course. Comparing the size of the coefficients, mechanisms acting through educational attainment appeared to be particularly important.

## Discussion

This study finds that parental separation is likely to have long-term implications for psychological health in adulthood which is consistent with previous studies e.g. [1-4,9,30,38]. We also found that this long-term impact of separation is translated via a complex chain of material and relational disadvantage across the life course akin to Amato’s ‘divorce-stress-adjustment perspective’ [37]. Sweeting and West [48] suggested that family of origin may be associated with adult health through an ‘unhealthy life career’. In particular they suggested children may set out on a path of disadvantage with respect to psychosocial and material factors, which subsequently increases their risk of poor health in later life. This finding is supported by this study. Education was found to be particularly important, suggesting that supporting children experiencing family breakdown through education may be one way to ameliorate the potential negative effect of parental separation. Additionally education was related to both material and relational pathways, suggesting that education does not solely reflect material factors but may have wider-reaching benefits.

This study finds that material and relational factors were interrelated across the life course, which extends previous work, particularly through the statistical approach taken to investigate how this might operate. For example material disadvantage in adolescence impacts upon parent–child relationships, offering additional support to Conger’s Family Stress model [33], which suggests that hardship puts strain on family relationships.

Previous work suggests that separation may have differing implications for men and women, and our study supports this [1,40]. To our knowledge this is one of the first studies to investigate whether explicitly-modelled pathways between parental separation and adult psychological distress differ by gender. The results of this study suggest that men’s, though not women’s, partnership status may be involved as a mediator. This is likely to be driven by lower marriage rates in sons of divorced parents combined with significantly poorer psychological health of single men. Additionally Fuhrer and Stansfeld [49] find that partnerships are not the first reported source of emotional support for women in the Whitehall II study, potentially identifying partnerships to be less important for women’s psychological health compared to men’s. Conversely the association between parent–child relationships and educational attainment was only important for women. This may be due to increased importance of parental involvement which may be more important for girls [50].

Around a third of the total effect of parental separation on psychological distress was accounted for by the indirect material and relational mechanisms for men. The remaining two-thirds, in standard path analysis terminology, would be described as a ‘direct effect’ of parental separation. This should be regarded as potentially due to measurement error or the involvement of additional un-investigated mechanisms. In total the material and relational mechanisms explained around 60% of the association for women.

It is only quite recently that both the necessary data and techniques to study the long-term effects of childhood adversities have become widely available. The techniques and data employed in this study give a better insight into why parental separation might be associated with increased psychological distress in adulthood. However this study is not without its limitations. Firstly no measures of parental conflict prior to parental divorce are available in this cohort. It is possible that parental conflict increases psychological distress in adulthood. However it is less plausible that it is conflict which influences material factors during childhood, such as adolescent material disadvantage. Adolescent material disadvantage is more likely to be influenced by the transition from a two-parent to a single-parent family. It is conceivable, though we cannot test it here, that parental conflict may operate through primarily relational pathways. Our results indicate that material pathways such as that working through low educational attainment, and subsequent living standards, may be more important in linking parental separation to adult psychological distress. However it is possible that material factors were captured with less measurement error than relational factors available in this dataset.

There are many important strengths of this study. Firstly the data used come from a large birth cohort with a large sample representative of the British population of a similar age. All measures were prospectively collected, therefore limiting recall bias, a potential problem in previous work. Secondly, missing data were accounted for using multiple imputation by chained equations, a method appropriate where data are missing in several variables. This method assumes that information is missing at random; that is, data are missing given observed values of other variables. In a large multidisciplinary dataset, such as this, the assumption is likely to hold and certainly be more appropriate than the assumption of missing completely at random which underlies a complete case analysis. Thirdly, we employ path analysis as a more suitable statistical method for the explicit modelling of pathways acting across the life course, therefore building upon previous work which has investigated mediation using multivariate regression methods unable to estimate multiple indirect pathways.

## Conclusions

In conclusion, using a large British birth cohort we find that parental separation is associated with increased psychological distress in adulthood for both men and women. This is partly explained by the complex linkage of material and relational mechanisms acting across the life course. This study highlights the importance of supporting separating families, both materially and relationally, in order to minimise long-term implications for psychological health. Interventions to break the link between parental separation and poorer educational attainment for both men and women, as well as initiatives to support high quality on-going parent–child relationships and material circumstances in families undergoing separation, may help achieve this.

## Abbreviations

A-Level: Advanced level; CFI: Comparative fit index; CSE: Certificate of secondary education; O-Level: Ordinary level; RMSEA: Root mean square error of approximation; TLI: Tucker Lewis Index.

## Competing interests

The authors declare that they have no competing interests.

## Authors’ contributions

RL designed the study, carried out the analyses and drafted the manuscript. MB, HP, MS and NC were all involved in providing input on the design of the study and drafting the final manuscript. All authors read and approved the final manuscript.

## Authors’ information

This work was completed as part of the ESRC International Centre for Life Course Studies in Health and Society.

## Pre-publication history

The pre-publication history for this paper can be accessed here:

http://www.biomedcentral.com/1471-2458/14/272/prepub
